# ‘Progression capitals’: How homeless health peer advocacy impacts peer advocates

**DOI:** 10.1016/j.socscimed.2022.114770

**Published:** 2022-04

**Authors:** PJ Annand, Lucy Platt, Sujit D. Rathod, Paniz Hosseini, Andrew Guise

**Affiliations:** a King's College London, United Kingdom; b London School of Hygiene and Tropical Medicine, United Kingdom; c University of Surrey, United Kingdom

**Keywords:** Peer support, Advocacy, Homelessness, Volunteerism, Voluntarism, Social capital, Recovery, Recovery capital

## Abstract

This article presents analysis from a qualitative evaluation of a homeless health peer advocacy (HHPA) service in London, United Kingdom. Whilst evidence is growing for the impact of peer programming on clients, understanding of the impact on peers themselves is limited in the context of homelessness. Research here is vital for supporting sustainable and effective programmes.

Analysis of interview data with 14 current and former peer advocates, 2 members of staff and 3 external stakeholders suggests peer advocacy and its organizational setting can generate social, human, cultural and physical resources to help peer advocates fulfil their own life goals. We explore these with reference to ‘recovery capital’, reframed as ‘progression capitals’ to reflect its relevance for pursuits unrelated to clinical understandings of recovery. Progression capitals can be defined as resources to pursue individually determined goals relating to self-fulfilment.

We find engagement with, and benefits from, a peer advocacy service is most feasible among individuals already possessing some ‘progression capital’. We discuss the value of progression capitals for peers alongside the implications of the role being unsalaried within a neoliberal political economy, and comment on the value that the progression capitals framework offers for the development and assessment of peer interventions more broadly.

## Highlights

• HHPA can support advocates to ‘recover’ from health issues and fulfil self-defined life goals.
• Reframing ‘recovery capital’ as ‘progression capitals’ better captures HHPA's impact on peers.
• Progression capitals are resources to pursue self-determined goals relating to fulfilment.
• Organizations should maximize the progression capitals such programmes can enable for peers.
• Progression capitals offer a framework for developing/assessing peer programmes across sectors.

## 1 Introduction

Research has shed light on the importance of peer advocacy and other forms of peer support interventions for individuals, communities and society (Stubbs et al., 2016, Lawton-Smith and Basset, 2013, Adams, 2020, Schwartz and Sendor, 1999, Locock and Brown, 2010, Kia et al., 2021). This literature explores health and social care outcomes for clients using peer advocacy services or other forms of “intentional peer support” (Bradstreet, 2006). It also highlights the benefits of such services for peers themselves, including positive impacts on employment and “educational preparedness” (Gammonley and Luken, 2001); improved self-esteem and health management (Repper and Carter, 2011); and “behavioural risk reduction” in contexts of drug use (Latkin et al., 2003). Challenges meanwhile include navigating the liminal space between health worker and service user (Scott et al., 2011) and consequent issues around independence, credibility, funding, flexibility and avoiding “red tape”; deploying experiential expertise while maintaining “professional boundaries” (Adams, 2020); and maintaining the right level of involvement in light of training received (Basset et al., 2010).

Despite the growing body of work on the impacts for peers of peer programming in the context of mental health, HIV, Hepatitis C and/or drug and alcohol support (Surey et al., 2019, 2021), few studies have explored peer service provision within homelessness (Barker et al., 2019), little of which focuses on the experiences of peers – as opposed to clients – in this context. The Barker et al. (2018), Cloke et al. (2007), Croft et al. (2013) and Miler et al. (2020) studies serve as notable exceptions here. These studies have drawn attention to issues of vulnerability regarding peer workers' own journeys and health; authenticity of experience, which some fear may be ‘formatted out’ by formal training; challenges around defining and maintaining appropriate boundaries with clients; stigma from having lived experience of homelessness; and limits on remuneration and career development. This work has also shed light on a number of significant benefits for peer supporters, including emotional satisfaction, self esteem, purpose, identity development, support with employment and training, and sustained sobreity for some with experience of issues around drug and alcohol (Barker et al., 2018). While such research has highlighted a number of key themes central to peer experiences, there is a need to build on these and develop theory (*ibid*.). We address this need by exploring whether and how homelessness peer programming can impact peer advocates themselves. We answer this question with reference to qualitative findings from a study into Homeless Health Peer Advocacy (HHPA) – a project run by London-based charity, Groundswell, which sees people with experience of homelessness helping those currently homeless to access health services.

## 2 Setting

Groundswell is a registered charity in London, UK, established in 1996, consisting of 18 staff and a variable number of volunteers. Approximately two thirds of staff have previous or current experience of homelessness themselves. The Homeless Health Peer Advocacy (HHPA) service run by Groundswell seeks to support people experiencing homelessness to access healthcare through the provision of trained peer advocates with experience of homelessness themselves (Groundswell, 2021b). The Groundswell peer advocacy model fits within a broader typology of peer involvement in health care processes (Dennis, 2003). It differs from the informal support people might give each other within hostel or street settings, or organized support groups, since it is unidirectional and intentional (Barker and Maguire, 2017; Bradstreet, 2006). Peer advocates support clients by accompanying them to appointments at health services (such as primary care or dentist appointments). They provide logistical and psycho-social support prior to appointments, facilitate communication and supportive interaction with health care providers, and relay information to key workers with clients' consent to facilitate integrated care. Secondary activities include awareness raising within street and hostel settings of care access opportunities (‘in-reach’). Peer advocates are volunteers, with some becoming (salaried) case workers after having successfully applied for advertised positions requiring peer advocacy skills.

## 3 Methodology

The qualitative analysis in this article formed part of a mixed-methods participatory evaluation of the HHPA project (Rathod et al., 2021). As such, people with experience of homelessness had key roles in shaping the design, delivery and dissemination of the research. The qualitative arm of the study had two aims: (a) explore how HHPA works, and (b) explore the impact of HHPA for peer advocates, the latter of which constitutes the focus of this article.

Data collection revolved around semi-structured in-depth interviews with current and former peer advocates (n = 14) recruited via Groundswell team meetings. Three of these had been appointed as caseworkers by the time they were interviewed, so reflected back on their volunteer experiences when responding to questions. Staff members (n = 2) and stakeholders (n = 3) were also invited to interview where it was felt that their perspective could add context to or be triangulated with the data provided by peer advocates, and were thus recruited purposively. Potential participants who expressed an interest in being interviewed were provided with an information sheet before being contacted by one of the research team to discuss the process and asked to complete a consent form.

Interviews were undertaken by a post-doctoral researcher experienced in qualitative interviews and analysis (PA), initially at Groundswell's offices (August 2019 – March 2020), and subsequently by telephone following the COVID-19 outbreak (March 2020 – December 2020). Six interviews took place in person and 13 were conducted by telephone post-March 2020. Two peer advocates who were active both before and during the pandemic were interviewed twice to explore experiences in more depth. Interviews followed a semi-structured interview guide consisting of questions exploring peers' experiences as a peer advocate, including their views on the training and support offered, the day-to-day details of their role, and their motivations for volunteering. Interviews lasted between 40 and 90 minutes and were audio-recorded and transcribed. We sought to interview as many peer advocates as possible, though stressed the voluntary nature of participation. We followed Groundswell's own policies for thanking participants, offering £10 as a token of appreciation. Data collection was additionally informed by immersion of PA within Groundswell between August 2019 – December 2021, including one course of in-person peer advocate training for prospective volunteers, and immersion of AG between 2017 – Dec 2021). This immersion within the organization's office spaces in Brixton (South London) and within virtual meetings when operations moved online during the pandemic facilitated understanding of the context of the service, thereby shaping the development of interview questions, and helped interviewees feel more comfortable during interview. This was especially vital as interviewers for this part of the study did not have lived experience of homelessness.

Data analysis was iterative to allow emerging theoretical insights to be integrated into ongoing data collection. We followed a grounded and abductive strategy (Green and Thorogood, 2014; Tavory and Timmermans, 2014) involving detailed coding of the data by PA and comprehensive second coding by AG. This took place alongside constant comparison and exploration of links across this coded data via analytical discussions between PA, AG, LP, SDR and PH with theoretical coding also incorporating broader social science scholarship throughout the analysis. Inter-coder agreement was achieved by consensus. Supportive analytical strategies also included memo writing by PA to explore conceptual ‘hunches’ and theoretical links, as well as periodic validation of findings with Groundswell staff and volunteers (Glaser and Strauss, 1967). The structure of this paper reflects the analytical process adopted – that is, one of descriptive but also theoretical coding. Accordingly, extant literature and theory that formed part of the analytical process, including that relating to capitals, is presented within the analysis itself.

## 4 Theoretical foundations

Notions of ‘recovery’ have long been central to efforts to conceptualize how people move beyond experiences of homelessness, and how that may link to mental health and drug use (Pahwa et al., 2019). Early uses of the term were rooted in the mental health liberation and psychiatric survivor movements (Frese and Davis, 1997; Ostrow and Adams, 2012, Mclean, 2000) which, alongside other civil rights campaigns of the 1960s and 1970s, “sought to question power and emphasized the importance of autonomy and self-determination” (Murray, 2019). In many settings today, conceptions of recovery have since departed from their earlier roots. In the UK, recovery has come to have many meanings, including concepts of clinical recovery – that is, “cure or remission of the illness” – and personal recovery – i.e. “functioning at one's best despite ongoing symptoms”, with the latter also incorporating wider social determinants of health (Barber, 2012, p. 277). Ideas relating to these various conceptions of recovery appear to be at the heart of Groundswell's work and how peer advocates describe their experiences of HHPA. As such, theories of recovery became central to our analysis, with the research team incorporating the recovery literature at the theoretical coding stage.

The concept of recovery *capital* stemmed largely from research into drug use, as well as mental health, as a way of framing “the breadth and depth of internal and external resources that can be drawn upon to initiate and sustain recovery” (Granfield and Cloud, 1999). In light of the divergent understandings of the meaning of recovery discussed above, critiques have emerged around how “the medical model of addiction and a pervasive culture of monitoring and performance targets” have led to a focus on basic quantitative indicators for recovery, defined by professionals without meaningful input from patients (Neale et al., 2015, p. 27). Such medical model conceptualizations, which emphasize “symptomatic improvements and functional status” as outcomes (Ahmed et al., 2012, p. 4) stand in tension with harm reduction approaches and may be considered at odds with earlier understandings of recovery within survivor movements. Other researchers lament the apparent neoliberal co-optation of the term recovery by government and public health bodies and its subsequent, narrower redefinition within mental health policy and services, for example, equating recovery with one's capacity to be productive (Frayne, 2019). In light of such commentaries, an initial overview of how such constructs are construed for the purposes of this article is prudent.

Firstly with regards to the notion of capital, we draw on Bourdieu (1986), emphasizing its inextricability from the interpersonal and external factors that shape experience and behaviour. We approach capitals as embedded in broader politico-economic and social structures and ‘fields’ (*ibid*.), which contribute to the radically unequal distribution of such capitals; this position contrasts with perspectives that shift responsibility for lack of capital, and associated outcomes, onto individuals (as described by Brown and Baker, 2012). Our analysis likewise resists the idea that a social system requiring the possession of capital for its effective navigation is necessarily a good or just approach to governance, while simultaneously acknowledging the realities of this prevailing political economy in the UK and beyond.

Secondly, recovery *capital* is generally thought to comprise four components: social (value of relationships), cultural (ability to project normative values and attitudes), human (personal traits that help someone prosper), and physical (finances or belongings) (Granfield and Cloud, 1999). While maintaining this basic structure, our analysis reframes recovery capital in terms of ‘progression’ – a term used within Groundswell itself. If recovery capital is the resource needed to facilitate a change in condition that satisfies institutional/external criteria (for recovery), then progression capitals – as we explore below – are those required for change according to *self-determined* criteria (for progression towards life goals).

## 5 Results

Our sample included 14 peer advocates across a range of demographics, including gender (60% female; 40% male), ethnicity (33% Black British/Black African; 33% mixed ethnicity; 33% white), educational experience (50% university; 33% vocational; 17% sixth form), eligibility for public funds (83% eligible), and Dis/abled identity (40% Disabled), as well as two staff members and three stakeholders (nurses and hostel staff who refer into HHPA) who were purposively selected. We deliberately withhold any more information to avoid indirect identification of interviewees.

### 5.1 Overview

**Fig. 1:**
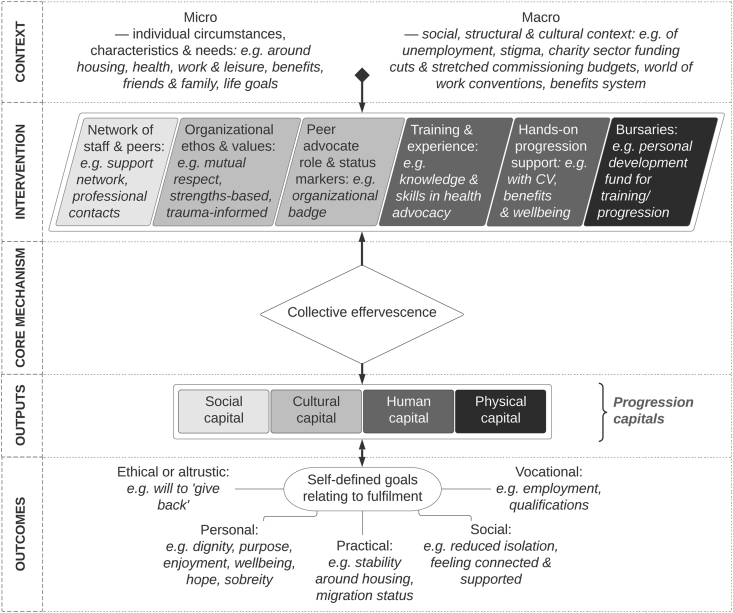
Summary of analysis

The analysis we present focuses on the impact of the HHPA project for peer advocates, along with the social processes that inspire such impacts. We first give a descriptive overview of how peers discussed the intervention and what it offered them, before exploring how peer accounts resonated with the concept of ‘recovery capital’ (a framework engaged with as part of the theoretical coding stage of analysis) but with limits. We then suggest the notion of ‘progression capital’ to better conceptualize how peer advocates sought to achieve self-directed goals around fulfilment rather than necessarily orienting their journey towards institutional or professional-defined indicators of recovery (albeit with the former sometimes overlapping with the latter). The section goes on to explore each component of progression capital in depth. We then elaborate on the mechanism of ‘collective effervescence’, found to be central to the development of these capitals, before reflecting on the voluntary nature of the peer advocate role. The analysis is summarized in Fig. 1.

### 5.2 Impact of being a peer advocate: progression

In a context of stigma, underemployment, health and wealth inequalities, housing precarity and other social injustices, the data suggest that being a peer advocate equips individuals with a range of useful resources and support. For example, peers stressed the importance of the support network they gained access to upon joining Groundswell:

*It's a very committed family … I've never seen such ethics … the network is very supportive, and they really make sure that they stick to the mission of what Groundswell was, was formed which is to take people out of homelessness.* (Peer Advocate, 2–3 years)

This support network was described as consisting of staff members at all levels, but also fellow volunteers, who peer advocates could speak to in times of need. In addition to this wider network, peers spoke of the significance of having contact with staff whose remit was to provide them with hands-on assistance navigating systems and processes needed to move through life, including help with benefits, wellbeing and job-seeking:

*[Staff member] helps you with your CV, and any training courses … they can write you letters on your behalf for DWP, or if you have any benefit problems, so yeah, it's awesome.* (Peer Advocate, 2–3 years)

Peers reported that this was complemented by the provision of bursaries for personal and professional development:

*If I need anything, anything regarding education they will fund you for that … they would get me a laptop, if I need a new laptop. So but for the one they got me, [it's] not a very expensive one but it's doing the job.* (Peer Advocate, <1 year)

Participants also discussed the value of the training and experience gained in the course of the HHPA role itself:

*It's fully comprehensive, you've got safeguarding training, first aid training … dealing with difficult behaviour, awareness of, you know, how to push wheelchairs, everything's in there. It's really good. Advocacy training, of course, because it's an advocate job. And we've got different people coming in to teach us different aspects.* (Peer Advocate, 2–3 years)

This was complemented by a degree of credibility and ‘status’ that some peers experienced by virtue of being a lived-experience expert and health advocate:

*We've got NHS lanyards and we've got NHS on our Groundswell ID and I think that that does give us, even though we're not professionally qualified apart from advocacy, that does give us a bit of weight.* (Peer Advocate, <1 year)

Importantly, these functions were seen as going hand-in-hand with a broader ethos of respect, solidarity and understanding, fostered throughout the organization:

*It's not like an us and them sort of situation. So I don't feel like paid staff are valued more than volunteers, in fact, I feel it's the other way round, in a sense. I feel like volunteers are valued more.*(Peer Advocate, 2–3 years)

Each of the elements described as central to HHPA's impact on peers were discussed by participants in terms of resources or support – whether social, personal, cultural or financial – which peers could use to advance their journey towards various goals.

*I don't think they're celebrated as much for helping the people that are working for us as well. People like myself, that have changed my life through Groundswell … they're picking that person up from the street later and helping them to become someone else.* (Peer Advocate, > 3 years)

As such, peer advocates described the impact of their HHPA involvement in ways that can align with conceptions of ‘recovery capital’ within the fields of drug use and mental health research (Granfield and Cloud, 1999). Indeed, some participants referenced the importance of HHPA for their clinical recovery:

*[HHPA] interferes with my tendency to use and stuff like that … When I first cleaned my act up … first off I was really good at like staying away from it. But [it gets difficult] as you start to settle down again … [HHPA] helps me … being responsible for being places and stuff like that … and taking people to wherever … it is important to me staying clean.* (Peer Advocate, >3 years)

However, many did not frame their involvement with HHPA as supporting ‘recovery’. Indeed, as one stakeholder commented, many people with experience of homelessness do not easily relate to this term given the connotations it carries of returning to previous, more favourable circumstances:

*Often in this setting, we talk about recovery … for many of the residents, they hated the term … they said [it] implies that life is really great and then actually things kind of fall apart a bit and now recovery [means] getting back to it … Our residents were like, “When I was born, things were terrible. I was witnessing domestic violence, I was abused. I'm not recovering for this great life.”* (Stakeholder, hostel keyworker)

Instead of necessarily referring to recovery, many peers discussed their HHPA experience as supporting ‘progression’ on their journey towards a broad range of goals linked to self-fulfilment. This terminology draws from the ‘progression’ function in Groundswell – a programme of “coaching to help volunteers and staff with experience of homelessness identify their goals and overcome … barriers” (Groundswell, 2021a). A dedicated team works with peer advocates in a range of areas, including support with debt, legal issues, benefits and career development to help them progress in self-determined ways:

*They have a progression manager that helps you with whatever situation you're in. If you need training, if you want to do some courses … if you have some housing issues, problems, etcetera … [it's] part of progression … So they're helping me [on] my journey.* (Peer Advocate <1 year)

While many professionals do include wider social determinants of health in their understandings of recovery, this is not always the case. Furthermore, it is still the case that goals and indicators for recovery tend to be defined without input from patients or communities (Neale et al., 2015). The range of objectives Groundswell supports peers to pursue, and the agency in determining them that peers describe, thus suggests that current usages and understandings of recovery and recovery capital may not be most apposite for conceptualizing the impact of HHPA, how peers experience HHPA, nor the principles and approaches the organization adopts. The term ‘progression capital’ – derived from the *in vivo* term ‘progression’ used within Groundswell – we argue, can more adequately incorporate the expansive ways peer advocates understand and deploy these constituent capitals, and the principles of trust and self-determination that underpin the organization's enabling thereof. If recovery capital is the resource needed to facilitate a change in condition that satisfies institutional/external criteria (for recovery), then progression capitals are the resources needed to pursue fulfilment in life according to *self-determined* criteria (progression).

### 5.3 How progression is enabled

We proceed by further elaborating on the idea of progression capitals in peer advocates’ accounts, highlighting the social, cultural, human and physical components described, and the HHPA functions that participating peers associated with each.

#### 5.3.1 Social capital: network of staff & peers

Social capital can be understood as resource available to someone by means of their relationships. Such relationships confer benefits and mutual obligations to individuals, who may then use this ‘stock’ to improve their circumstances (Cloud and Granfield, 2008). Peers' HHPA participation provides access to such social stock, in return for their commitment to the role, with one peer advocate explaining that “*the more you give, the more you get back*”. Volunteers give their time to deliver client-focused work, administrative tasks and training. These obligations are accompanied by an expansive social network from which peers appear to draw significant social resource:

*I can talk to anybody in the office … they'll take the time to talk to you … from CEO right down to a fellow volunteer.*(Peer Advocate, 2–3 years)

The community of staff and volunteers serves as a source of information, support and opportunity for peer advocates. Peers can draw upon this resource and direct it towards self-fulfilment goals, for example, via professional development opportunities made available:

*Being involved in research projects myself … wouldn't have happened if I wasn't here … things like that, just … opportunities … I think if HHPAs have their eye out and it's something that they would like to do, they can … When I am ready to go back into the employment field, I think I'll have a lot of things on my CV that'll be looked upon favourably.* (Peer Advocate, 2–3 years)

Moreover, being linked in with support staff at Groundswell – the ‘progression team’ – similarly serves as an opportunity for peer advocates to access one-to-one assistance with resolving personal problems, professional concerns and other issues that may arise in the course of life.

*That support network that takes the pressure of that [personal circumstance] off you so you can do this job and then build yourself up. And I use myself as an example like I've got a career now.* (Peer advocate, 2–3 years)

Thus for some, such assistance makes space for individuals to fulfil further outcomes, such as any employment they may wish to pursue.

#### 5.3.2 Cultural capital: peer advocate role and status markers

Cultural capital derives from an individual's familiarity with normative (especially ‘high culture’) values, beliefs and attitudes, and the ability to project cultural signals that convey affinity with these norms (Bourdieu, 1984). As such, cultural capital largely pertains to a capacity to ‘fit in’ with dominant social behaviours. The stigma attached to homelessness means that people experiencing it can lack the cultural capital from which others may benefit to access and navigate social settings and systems. This paves the way for, and is exacerbated by, systems that are not set up to listen to or accommodate the needs of people affected by homelessness – described by one peer advocate as “*designed-in exclusion*”. Being able to demonstrate familiarity with the complaints system within the National Health Service (NHS) – gained through training and experience in the course of being a peer advocate, or indeed sometimes before – along with a sense of credibility that comes with the HHPA volunteer status itself, can be said to confer cultural capital. As one interviewee noted, when there is an issue, “*we go there to talk to the doctor, because we have this badge, doctors know, know we can put a case against them*”. Cultural capital thus may be conveyed by visual cues such as peers' badges and NHS lanyards. Some interviewees pointed to how this helped improve their confidence generally, thus contributing to the development of human capital (explored further in the next section).

Here, it should be noted that cultural capital may change according to the ‘field’ (Bourdieu, 1984) or social sphere, to include for example one's reputation or ability to demonstrate credibility within a particular group. The cultural capital associated with homelessness, Barker (2012) suggests is acknowledged but ultimately judged negatively by broader society and, as such, frames it as ‘negative cultural capital’. However, Groundswell carves out a space where such alternative forms of cultural capital are valuable. For peers, the cultural capital available to them by virtue of their lived experience of homelessness, and familiarity with the field, is precisely one of the resources most prized by the organization:

*I thought I had nothing other than a … driving license … but I had my wealth of experience and Groundswell [highlighted] that.* (Peer Advocate, >3 years)

This approach, in many ways, reflects notions of ‘critical resilience’ (Monforte, 2020): the HHPA project does not take the system as it is and simply help those with experience of homelessness navigate it better, or become more resilient to its effects, it also challenges it by trying to create spaces where such norms are resisted in favour of more inclusive values.

#### 5.3.3 Human capital: hands-on support

Human capital refers to the “acquired or inherited traits” that enable individuals to prosper in a given society, such as skills, knowledge, educational credentials and good health (Cloud and Granfield, 2008, p. 1974). The relevance of human capital for peer advocates relates, broadly speaking, to two principal functions that can be characterized in terms of hands-on support for peer advocates. The first of these revolves around the training and development offered to all peer advocates via the progression programme:

*I could go to my HHPA Project Manager and say, I'd like to be trained in research. And I've got a progression worker as well … we check in once a week … [and] look at opportunities for the future.* (Peer Advocate, <1 year)

This goes hand-in-hand with advocacy training and expertise gained in the course of peers’ roles. Peer advocates link this training and support with outcomes such as upskilling, developing knowledge and understanding, and gaining experience – that is, acquiring human capital.

The second pertains to the one-to-one and group clinical supervision/reflective practice offered to all volunteers. Group sessions take place on a fortnightly basis with a dedicated counsellor, and volunteers can also arrange one-to-one sessions. Participants described these sessions positively, with many discussing its benefits for their own emotional wellbeing:

*[One way] Groundswell look after the staff is the clinical supervision … Really brilliant. People pay a lot of money for that and our clinical supervisor is fantastic … If we have issues, such as triggering, we can bring it there … we can have a one-to-one with the clinical supervisor as a one-off and say, “Look, this is really bothering me, can we meet and talk?”* (Peer Advocate, 2–3 years)

Furthermore, resource to support mental health and emotional wellbeing was described as emanating from the community and social support network:

*That's the benefit of being in Groundswell … feeling supported, and someone to talk to … if you're depressed, you can phone up … [or] pop in and have a chat with someone.*(Peer Advocate, <1 year)

Peers discussed being able to draw upon this formal and informal support to help them through difficult times, with outcomes relating to human capital being reported, including decreased feelings of isolation.

#### 5.3.4 Physical capital: personal-professional development bursary

Finally, we turn to our attention to physical capital (Cloud and Granfield, 2008), which are material assets such as money and a place to live. While the peer advocate roles are not salaried, Groundswell does provide its volunteers with access to certain forms of physical capital. As well as out-of-pocket expenses associated with travel and meals, peers can access funds to assist them with skills development or other progression goals:

*There's a bursary once you've been with Groundswell … a certain amount of time which can help toward something to do with progression. I'm using mine to help get a laptop that helps me with my studies and things … They look after the staff anyway in terms of covering expenses, which most … charities do, but I think they're quite good at doing it.* (Peer Advocate, 2–3 years)

Peer advocates can use this physical capital for a course, equipment, or as one peer advocated described it, “*buying something nice*”. This is reported to be a resource of significant value to volunteers, sometimes serving as a motivation to persevere with training and become a peer advocate in the first place. Indeed, some peer advocates believed that one way to attract and retain new volunteers, especially during the COVID-19 pandemic, which saw the number of active peer advocates diminish, may be to offer the bursary (or alternative funds or even remuneration) earlier in the volunteering journey.

### 5.4 Pre-existing capitals needed for progression

It should be noted that while Groundswell's social, cultural, and human resources are offered in different ways and for different purposes throughout the lifecycle of peer advocates' volunteering journey, they are generally all immediately available, in some form, from the first stage of training. Physical capital, however, represents a resource that is perhaps less obviously available from the outset, as it is only after having volunteered for long enough to have discussed, developed and devised progression goals and pathways that bursaries are made available to peer advocates. However, some trainees leave before having completed training, thus halting access to its rewards. Some individuals simply realize that they no longer want to volunteer or that the role isn't the type of endeavour that suits them, yet for others there is reportedly a desire to continue which ultimately goes unrealized.

*Some people … they've got mental health or … sometimes it just ends up too much … it's not easy if you've got lived experience … it can be triggering … it's just at what stage people are in their lives.* (Peer Advocate, <1 year)

*You must be in stable accommodation and … if you've got an addiction that must be under control, so those sorts of things could prevent someone from volunteering.*(Peer Advocate, 2–3 years)

Such data provides insight into the question of for whom (i.e. what type of prospective volunteer) HHPA can be considered most viable, and for whom it might have impact. Indeed, that which appears to distinguish between those who complete training and those who do not relates to a quality that many participants described as ‘readiness’ – for example, in terms of housing (a form of physical capital), mental health or literacy (forms of human capital). In this article, we frame these as the progression capitals available for them to draw upon. Namely, those with more progression capitals, especially in the form of physical and human capital, represent those most likely to succeed in the peer advocacy endeavour, thus in turn benefitting from the resources HHPA offers. Meanwhile, for those lacking in the levels of progression capital or ‘readiness’ (potentially those who could benefit from such resources the most), peer advocacy may not be an effective, or viable, means by which to gain the resources they need to progress.

### 5.5 Collective effervescence as a mechanism for progression

The analysis presented so far has posited that involvement in peer advocacy enables the development of social, cultural, human and physical capital, and that those who complete the training process and become peer advocates are most likely those arriving with an existing level of progression capital. In this section we explore a key mechanism facilitating progression capital among peer advocates (and the lack thereof among some of those who do not complete the training process), which pertains to the emotional and interpersonal energy generated by being part of the Groundswell community. Our immersion in Groundswell's offices enabled us to observe some of these more intangible characteristics of the project, providing a foundation for exploring this element further in interviews thereafter. The interpersonal energy to which we refer could be identified in the organization's offices at large, but was also demonstrated clearly in the HHPA training environment. It was here that prospective volunteers congregated, initially as strangers, to learn the skills necessary for becoming a peer advocate. In addition to the development of skills, however, a key part of the training process involved the facilitator crafting a safe environment within which trainees could co-create shared values, develop mutual trust and respect, and gradually build bonds that allow for greater self-expression and vulnerability:

*You're walking in and you're telling them all the things which most places you don't tell people that, you tend to keep yourself to yourself … and they're like same here, we're doing this one, and you're like, oh my God yeah … it makes everything seem very normal … it was just brilliant being in there.* (Peer advocate, <1 year)

When exercised, these generated positive emotional responses that, in turn, appeared to sustain and reinforce the integrity of the group and the relationships that constituted it.

This interpersonal energy bears resemblance to Durkheim's (1912/1995) concept of ‘collective effervescence’, which can be summarized as the “affective arousal of an assembled crowd [that] creates the potential for both social conformity and group-based agency” (Liebst, 2019). While HHPA trainee cohorts, which rarely comprise more than 15 people, are much smaller than common understandings of a crowd, similarities may be identified with regards to affective arousal and the simultaneous functions of conformity and agency. For example, while prospective HHPA volunteers ostensibly seek to develop, promote and conform to a set of shared values around mutual respect and non-judgement, at the same time, a sense of group-based agency can also be identified in their ambition to collectively realize broader goals around improved health for those who are homeless. These functions appear to be fuelled, at least in part, by a form of interpersonal energy similar to collective effervescence, generated through the experiences and bonds that prospective advocates share in the training environment and, later, in the Groundswell offices.

*Being altruistic and helping others, that's … now that's what I get out of it, but at first I didn't really see that side … But it happened through Groundswell, meeting everyone here, I suppose being kind of infected or I suppose that nature of people is contagious, you know?* (Peer advocate, >3 years)

This participant discussed the interpersonal energy generated within Groundswell in terms of contagiousness. Others described it as an energy or atmosphere that ‘clicks’:

*I came in [to Groundswell], and like it just clicked, and I keep saying to everyone it felt really, really cheesy, but I've never ever walked into a building … of work and never thought no I actually want to work here, like before even really knowing what Groundswell was.* (Peer Advocate, <1 year)

Such accounts resonate with broader understandings of collective effervescence as an interactional effect triggered by an ‘emotional contagion’ (Collins, 2004) whereby “interacting participants influence and intensify each other's emotional state” (Liebst, 2019).

While not ritualistic in the sense of bodily synchronicity and entrainment, prospective advocates attend training three times a week over a period of one month, where they engage in guided activities. Upon ‘graduation’ this is followed by regular volunteer meetings, clinical supervision sessions, progression support meetings and client appointments. These can perhaps be understood in terms of Collins (2004) ‘interaction rituals’, which may not reach the energetic heights of collective effervescence, but nonetheless are able to create significant, positive interpersonal energy.

Indeed, the importance of this type of collective process was highlighted when COVID-19 disrupted these rituals. Such energy was difficult to sustain when socially distancing and all meetings had to take place online:

*Communication [between] peers … is probably as good as it’s going to get with daily coffee breaks that anyone can kind of drop-in [online] … and there've been … quizzes and stuff … but then if [other volunteers] are not in the coffee breaks there's very minimal communication … with peers. With staff … there is also communication outside of that, particularly around work stuff, emails and things like that … and then also with the welfare checks that are being done by staff, which is really good … [The difference is] it's not varied … it's not like different people you have contact with, it's the one person who would be calling you each week* (Peer Advocate, 2–3 years).

Following this disruption during the pandemic, volunteer engagement could be seen to diminish. As the excerpt above suggests, however, reduced participation is not for lack of effort on the part of staff to maintain social contact and team participation during this era of physical distancing, but may instead be due to the greater ease with which Groundswell's ‘infectious energy’ can flourish in a shared *physical* space characterized by regular, face-to-face, social interaction rituals with a wider community of peers.

Returning to the idea of peers needing to arrive with a certain ‘stock’ of progression capital, of additional relevance here is Collins' assertion that only those with a degree of existing resource will tend to engage with such interaction rituals. If someone's personal resource or capital does not ‘match-up’ with the level of resource required to engage, it is argued, “the interaction ritual does not reach a high level of intensity, and the EE [emotional energy] payoff is low. Individuals are motivated to move away from such interactions” (Collins, 2004, p. 151). This resonates with participant accounts of who ‘makes it’ as a peer advocate and who ‘drops out’ before completing training. As discussed in the previous section, the latter are described in terms of their lack of readiness to become a peer advocate; a role which requires a certain degree of time, headspace and consistency, and thus a minimum level of human and physical capital. On the other hand, it seems those who are more settled, with greater human and physical capital, describe being more available for participation in – and ‘infection’ by – the emotional contagion of the peer advocate community, and thus more able to access any benefits it may lend.

### 5.6 Social context for HHPA: volunteerism and the pursuit of progression capitals

While the positive experiences of peer advocates at the individual level are apparent, it must be noted that the Groundswell community is not described as *uniformly* ‘progressive’ and some peers also expressed critical perspectives – as can perhaps be expected in any organization with a diverse body of staff and volunteers. In particular, some peer advocates articulated a desire for the role to be remunerated. Relatedly, the challenging socio-political environments that volunteers inhabit – and how these are reflected in their formulations of progression goals sought via engagement with HHPA – must be acknowledged and considered as part of the analysis. That is, the pursuit of progression capital via an unremunerated volunteering scheme cannot be divorced from the context in which such a pursuit becomes necessary.

Peer advocates expressed a range of reasons underlying their decision to volunteer. Some advocates discussed a desire to have more autonomy than paid work generally offers, either because they saw their involvement in HHPA as more of a leisure activity (akin to traditional understandings of volunteering and charity) or because their health and emotional wellbeing prevented them from being able to take on the responsibility, duties and time-commitment of full-time employment:

*There are roles for permanent staff and there's a role for the volunteer. [As] a volunteer, the manager will ask me … morning or afternoon shifts? So mostly I was comfortable with afternoon shifts because morning I may arrive late, because … with my health issues I had to see the doctor sometimes, I have to also have a rest, because was also homeless … [As a volunteer] you could be allocated whatever time you're comfortable with.* (Peer Advocate, 2–3 years)

Volunteering allows peer advocates to take up – or refuse – tasks at will and set their own hours. This may not be possible if the role were salaried, due to the difficulties in achieving flexibility around duties and hours within paid employee-employer relationships (relationships that are often based on hierarchies and ‘chain-of-command’). These reflections in many ways speak to doubts, also raised in the extant literature, regarding whether “the institution of employment can realistically cater to wellbeing” (PSYCHOLOGISTS-FOR-SOCIAL-CHANGE, 2019). Indeed, the need for, and benefits of, autonomy and control over the day-to-day realities of one's life is thought to be particularly strong among those with experiences of trauma (Slattery and Goodman, 2009) – common among those affected by homelessness (Goodman et al., 1991).

While Groundswell adopts a markedly trauma-informed approach in its ways of working, some degree of professional hierarchy is nonetheless in place. (We note here the challenges that have been identified around flattening organizational structures within the health and third sectors, where some degree of hierarchy is felt to support effective service provision (DiPalma, 2004)) Thus, for some advocates, the voluntary nature of the HHPA role suits their preferences and health needs enabling them to achieve quality-of-life goals around autonomy and flexibility. Such perspectives inevitably raise questions about whether alternative models of work may be imagined that allow for this labour to be paid (or for individuals to otherwise gain financial security and stability), while still maintaining the sense of autonomy and empowerment that goes hand-in-hand with volunteering.

Echoing similar findings from other peer studies (Allan, 2019; Miler et al., 2020; Cloke et al., 2007) some advocates speak of wanting to achieve fulfilment through “*giving back*”. This particular reason is bound up with the view that volunteering takes on an additional moral dimension that may be compromised if such activities were paid:

*As a volunteer, it sounds quite silly but … I sometimes feel like I've got the moral high ground … it's not something I get paid for, it's something that I really believe in and … I'm not too sure I could … do it [as] a paid role.* (Peer Advocate, >3 years)

For some peers, such beliefs also go hand in hand with assertions that HHPA works best as a volunteering (rather than salaried) programme of work. This reflects the notion that “money drives out love” (Chua and Clegg, 1990, p. 148) – a perspective that suggests care work is best performed when workers are motivated by doing good rather than earning money. While seemingly prevalent, such views have nonetheless been problematized given their tendency to then devalue and underpay care work (Overgaard, 2019).

Meanwhile, some peer advocates speak of a desire to get a paid job, which may be even more challenging in the context of high unemployment, elevated competition for roles, and stigma against people with a history of homelessness, drug use, mental health problems or contact with the prison system. These volunteers see HHPA as potentially opening up new career paths, and a possible stepping-stone to long-term employment:

*If they can make it a paid role … that's better. But at the end of the day … voluntary work is still making a very huge difference … Because that is one area I [am] seriously now considering to also make my career, properly.* (Peer Advocate, 2–3 years)

Groundswell's own figures show that many volunteers, who wish to, do in fact progress to salaried positions (sometimes within the organization itself). The extant research likewise suggests that volunteering does improve people's chances of finding a job (Spera et al., 2015) and moving beyond experiences of homelessness (Groundswell, 2008) – and such research was cited by staff interviewees as an evidence-based rationale behind the volunteering model.

However, more recent studies have questioned the conclusion that volunteering is necessarily the best route to permanent, full-time employment, given that much of the existing research has compared outcomes only between those who have and haven't volunteered – rather than, for example, comparing outcomes between those who have engaged in volunteering work versus work that is freelance or otherwise paid (Overgaard, 2019). Such literature challenges academic and policy researchers to ask “Why would anybody work for no pay *rather than for pay?*” instead of “Why do people volunteer?”. The latter Overgaard (*ibid*., p. 137) frames as “a question that seems to imply that the choice is between volunteering and lying on the couch” going on to assert that “it should now be possible to appreciate that ‘choice’ is a slippery notion and that access to *paid* work is at the heart of understanding unpaid work patterns”. Indeed, a number of studies suggest that meaningful paid employment can lead to significant improvements in health and employment/social outcomes for marginalized populations such as people who use drugs and people living with HIV, including those affected by homelessness and other structural inequalities (Richardson et al., 2012, 2013; Closson et al., 2016). This is a proposition that we have taken seriously in our own analysis, and have sought to address in this article.

## 6 Discussion

The impacts of being a peer advocate described in this article revolve around progression capitals, which peers appear to have developed further through their engagement with the HHPA project. These include the social capital generated by the relationships afforded them upon joining the team; cultural capital gained via the organization's approbation of lived experience, and provision of health advocate status; human capital via extensive training and clinical support; and physical capital via a bursary scheme.

Framing such resources as progression capitals, rather than relying on notions of recovery capital that are more commonly used within health and social care settings, helps us account for the ways that: (a) clinical recovery from health conditions (and other definitions of recovery used by professionals and institutions) is not always the main end goal, though this may represent a means to self-fulfilment for some; (b) the term recovery itself is slippery (Neale et al., 2015) with current usages potentially meaning little to some, including those without any history of drug use or health issues associated with their experience of homelessness; and (c) definitions of recovery, even those incorporating broader ‘personal recovery’ characteristics, arguably imply a return to previous more favourable circumstances, which those with long-term experiences of disadvantage may find difficult to relate to. A move away from conceptions of recovery capital towards understandings that align with progression capitals – so named to reflect the participants' and organization's own terminology – facilitates the recognition of (and emphasizes the validity of) individually-determined self-fulfilment goals. These may include – but are not restricted to – clinical criteria for recovery. At the same time, this reorientated understanding of social, cultural, physical and human resource in the context of homelessness allows us to resist any tendency to rely principally on measures of an individual's productivity or productive capacity to assess a service's efficacy.

## 7 Limitations

A key limitation of this study revolves around its scope, which did not include interviews with (a) trainees who had not completed the HHPA training course, (b) former peer advocates no longer working at Groundswell, and (c) people who knew about HHPA but decided against volunteering. Since the data suggested that some peer advocates volunteered as a means to paid work within a societal context of inequality and under-employment, and some expressed an interest in being remunerated for their peer advocacy work, we sought to engage extensively with the critical literature on volunteerism and peer interventions. We took seriously the concerns raised and incorporated this extant social science insight into our analysis. Other challenges that may relate to the HHPA context include those of burnout and work pressure, however the data we collected did not engage with these issues. Future research may wish to further explore the perspectives of non-volunteers and/or other settings where burnout and pressure may be more prominent in volunteer accounts, to build on our analysis (of whether and how peer advocacy has impact for those who *do* volunteer) by incorporating further insight into who peer advocacy does *not* work for and/or does *not* reach.

## 8 Conclusion

While social, cultural, human and physical capitals have tended to be invoked as resources for the purposes of recovery from issues relating to mental health and/or drug use, we argue these may in fact represent useful ‘stock’ for many people and communities striving towards a range of ends. We have suggested in this article that the resources often understood as recovery capital may, in the context of the HHPA project, be better understood as progression capitals. What HHPA offers is not just a way for those with experience of, for example, mental health issues to recover – in terms of common clinical understandings or productivity-related indicators thereof – or even in broader terms of a return to more favourable circumstances. Rather it is a means for those affected by homelessness and its many intersections to progress towards self-determined goals relating to self-fulfilment, in ways that may or may not include recovery indicators used in institutional settings.

Peer advocates represent individuals with diverse needs and wishes but who must operate within a political economy that requires them to possess capital to move forward. Arguably, however, as people affected by homelessness, they are also among those least likely to have access to it. The HHPA project, we argue, enables volunteers to access social, cultural, human and physical capitals, facilitating individual progression within existing structures (while simultaneously questioning and, in some ways, challenging the norms and values upon which these systems rely in the first place).

The organization studied, Groundswell, enables these progression capitals by means of its community and network; personal development team; bursary; training; clinical supervision; and trauma-informed ethos. While there may be alternative ways for peer initiatives to support their staff members and/or volunteers, our analysis suggests that attention to how such schemes can enable progression capitals will be an important consideration when seeking to maximize benefit for workers with experience of homelessness, including though not limited to possible benefits relating to mental health and drug use.

The theory of progression capitals presented in this article thus, we contend, serves as a useful framework for conceptualizing the potential impact of homeless health peer advocacy on peer advocates. Equally so, however, we also wish to conclude by pointing to the value this framework may hold for the purposes of assessing the impact of peer programmes on volunteers and staff with lived experience of homelessness (and, indeed, other forms of marginalization) within neoliberal political economies more broadly.

## Author contributions

**PA**: Investigation, Data curation, Formal analysis, analysis discussion, Writing – original draft, Writing – review & editing, Project administration; **LP**: Conceptualization, Funding acquisition, Supervision, Writing – review & editing; **SDR**: Writing – review & editing, analysis discussion; **PH**: Writing – review & editing, analysis discussion; **AG** – formal analysis, Data curation, Funding acquisition, Supervision, Writing – review & editing, Project administration.

## Funding acknowledgements

Research funded by National Institute for Health Research (NIHR) – Public Health Research programme (Award ID: 17/44/40). Gold Open Access funded by University of Surrey.

## Ethical approval

Dulwich Research Ethics Committee(IRAS project ID 271312) and King's College London Ethics Committee (REF HR-18/19–11,523).

## Declaration of competing interest

None.
